# In-situ study of electrochemical migration of tin in the presence of bromide ion

**DOI:** 10.1038/s41598-021-95276-0

**Published:** 2021-08-03

**Authors:** Ee Lynn Lee, A. S. M. A. Haseeb, Wan Jeffrey Basirun, Yew Hoong Wong, Mohd Faizul Mohd Sabri, Boon Yew Low

**Affiliations:** 1 Department of Mechanical Engineering, Faculty of Engineering, Universiti Malaya, 50603 Kuala Lumpur, Malaysia; 2 Centre of Advanced Materials, Faculty of Engineering, Universiti Malaya, 50603 Kuala Lumpur, Malaysia; 3 Department of Chemistry, Faculty of Science, Universiti Malaya, 50603 Kuala Lumpur, Malaysia; 4 Nanotechnology and Catalysis Research Centre (NANOCAT), Institute for Advanced Studies, Universiti Malaya, 50603 Kuala Lumpur, Malaysia; 5 Centre for Energy Sciences, Faculty of Engineering, Universiti Malaya, 50603 Kuala Lumpur, Malaysia; 6 Process Innovation, NXP Malaysia Sdn. Bhd., 47300 Petaling Jaya, Selangor Malaysia

**Keywords:** Chemistry, Engineering, Materials science

## Abstract

The miniaturization of electronic devices and the consequent decrease in the distance between conductive lines have increased the risk of short circuit failure due to electrochemical migration (ECM). The presence of ionic contaminants affects the ECM process. This work systematically investigates the ECM of tin (Sn) in the presence of bromide ions (Br^−^) in the range of 10^−6^ M to 1.0 M. Water drop test (WDT) was conducted in the two-probe semiconductor characterization system under an optical microscope as an in-situ observation. Polarization test was carried out to study the correlation between the corrosion properties of Sn and its ECM behaviour. The products of ECM were characterized by scanning electron microscope coupled with an energy dispersive X-rays spectrometer (SEM/EDX) and X-ray photoelectron spectrometer (XPS). The results confirm that the rate of anodic dissolution of Sn monotonously increases with the Br^−^ concentration. However, the probability of ECM failure follows a normal distribution initially, but later increases with the Br^−^ concentration. The main products of the ECM reactions are identified as Sn dendrites and tin hydroxide precipitates. The mechanisms of the ECM process of Sn in the presence of Br^−^ are also suggested.

## Introduction

The increasing demand towards miniaturized electronic devices has led to the development of higher density electronic packages with smaller components. The increasing number of leads and closer spacing between them have made electronic devices more vulnerable to corrosion related damages^1,2^. During manufacturing and in service, these devices are exposed to high humidity which leads to the formation of a corrosion cell. The reduced distance between the conduction lines due to a smaller pitch accelerates the corrosion process. Consequently, the life span of devices is reduced, sometimes experiencing catastrophic failure.

Electrochemical migration (ECM)^3–7^ is one of the corrosion related problems encountered in electronic devices. ECM is an electrochemical reaction which occurs in the presence of electrolyte and applied potential difference. The electrolyte results from the adsorption or condensation of moisture which connects the two conduction lines in the presence of contaminants. Under the influence of a bias voltage, the conduction lines become two oppositely charged electrodes. The corrosion or the dissolution of the conductive metal anode releases the metal ions into the electrolyte. This is followed by the migration of the metal ions from the anode to the cathode. The dissolved metal ions are electrodeposited at the cathode as dendrites, which propagate through the electrolyte and connect the two electrodes. This eventually leads to short-circuiting, which is a catastrophic failure in electronic devices^7,8^.

In response to the prohibition of lead (Pb) containing materials by the Restriction of Hazardous Substances (RoHS) Directive, pure tin (Sn) is widely adopted for the surface finishing of electronic devices^9^. Meanwhile, Sn-based Pb-free solder alloys are widely employed as solder interconnects in electronic devices^2,10,11^. A large part of the directly exposed interconnects on the printed circuit boards (PCB) consists of Sn and its alloys, but worst of all Sn and Sn solder alloys are susceptible to ECM^5,7,12^. This problem has posed great challenges to the reliability of electronic devices. Thus, the investigation into ECM of Sn is crucial for the electronics industry.

ECM can be categorized into humid ECM and condensed ECM^1,13^. Humid ECM occurs when a thin invisible moisture film is adsorbed on the surface while condensed ECM occurs in a visible layer of condensed water. The risk of condensed ECM increases when the electronic device is exposed to temperature fluctuations due to the difference between the device interior conditions and external climates^14^. The presence of hygroscopic contaminants facilitates water condensation on the surface and increases the electrolyte conductivity. These contaminants accelerate the corrosion process and are expected to promote the ECM. Ionic contaminants can arise from service environment, human handling or from the manufacturing processes^1,15^ such as the assembling process^12^. The most commonly found ionic contaminants in electronic devices are the halides and sulphates (SO_4_^2−^)^7,12^.

Previous reports^1,5^ showed that higher chloride (Cl^−^) concentration results in a higher anodic dissolution rate of Sn from 10 to 1,000 ppm. However, the probability of dendritic formation due to ECM decreases in the presence of Cl^−^^1,5^. This is attributed to the formation of excessive tin hydroxide precipitates which hinders the migration of charged ions at higher concentrations^16^. However, a later study found that an increase in the Cl^−^ concentration beyond 0.07 M (~ 4,091 ppm) leads to the re-dissolution of the precipitates and the subsequent dendritic deposition at the Sn cathode^17^.

Medgyes et al.^18^ varied the concentration of SO_4_^2−^ from zero to saturated concentration (~ 1.86 M) to study its effects on the ECM of pure Sn. The ECM lifetime was the shortest in lower concentration of SO_4_^2−^ (0.1 mM). Similar to the results reported in Cl^−1,^^5,16^, the intermediate concentration (10 mM) of SO_4_^2−^ caused only the formation of precipitates instead of dendritic growth^18^. From previous studies^1,5,15–20^, the presence of ionic contaminants in high concentrations does not necessarily lead to the increase in ECM failure, even though the corrosion of anode is more favourable at higher concentrations.

The relationship between the corrosion properties and the ECM lifetime of Sn-based solders is elucidated in the literature^21–25^. Yoo et al.^21,22^ found that the corrosion rate (I_corr_), corrosion potential (E_corr_) and pitting potential (E_pitt_) of Sn-based solders are affected by the addition of alloying elements such as Pb and bismuth (Bi). They reported that the time-to-failure (TTF) of the Sn-based solders increases with E_corr_ and E_pitt_ in 0.001 wt.% sodium chloride (NaCl) and 0.001 wt.% sodium sulphate (Na_2_SO_4_). However, the TTF is poorly related to the I_corr_ of the solders^21,22^. Jung et al.^23,24^ compared the TTF of Sn-based solders in 0.001 wt.% NaCl and 0.001 wt.% Na_2_SO_4_. They reported that the TTF is higher in 0.001 wt.% NaCl than in 0.001 wt.% Na_2_SO_4_ due to the higher E_pitt_ of Sn-based solders in 0.001 wt.% NaCl. Liao et al.^25^ reported that the addition of 1–500 mM tri-sodium citrate dehydrate into 1 mM NaCl effectively inhibits the growth of Sn dendrites due to the increase of E_pitt_ of pure Sn. In general, the ECM of Sn-based solders in the presence of ionic contaminants are suppressed when the E_pitt_ is higher and is not directly affected by the I_corr_^21–25^.

Bromide (Br^−^) is another common ionic contaminant found in electronic components^26^. The presence of Br^−^ is due to the utilization of halides such as hydrogen bromide (HBr) and carbon tetrabromide (CBr_4_) in the etching process during chip fabrication^27,28^ or the use of brominated flame retardants (BFR) in printed circuit board (PCB) laminates and in moulding compounds^29^. Recently, there is a necessity to phase out the use of BFR due to its harmful effects on humans and the environment^30^. However, RoHS still allows the presence of certain types of BFRs such as polybrominated biphenyls (PBB) and tetra-bromobisphenol A (TBBA) up to 1000 ppm, as effective flame retardants in electronic devices^30,31^. The degradation of BFR remnants in the electronic components subsequently releases the Br^−32^.

At present, most investigations on the ECM of electronic devices are focused mainly on the effects of Cl^−^ and SO_4_^2−^. The information on the effects of Br^−^ on the ECM process is still scarce. The effects of Br^−^ concentration^5^ from 10 to 250 ppm (equivalent to 8.4·10^−5^ M–2.1·10^−3^ M) on the ECM behaviour is the only report on the effects of Br^−^ on the ECM of pure Sn. The probability of ECM failure initially increases with the Br^−^ concentration but later decreases with further increase in the Br^−^ concentration. It was suggested that the further increase in the Br^−^ concentration beyond 250 ppm does not favour dendritic growth as higher concentration leads to precipitate formation without dendritic growth^5^. However, previous work^17,20^ reported that the precipitates could re-dissolve in extremely alkaline environment due to the presence of higher concentration of halide contaminants. This consequently leads to the dendritic deposition at the Sn cathode.

The effect of ionic contaminants on the ECM behaviour is rather complicated. Higher concentration of contaminants may or may not favour dendritic growth, but depends on the type of contaminants and mechanisms involved^1,5,17,20^. Correlation between the ECM behaviour and the corrosion properties is also complex^5,21,22^. This work attempts to obtain a comprehensive understanding of the mechanism of ECM in a wide range of Br^−^ concentration (10^−6^ M–1.0 M, equivalent to 0.1 ppm–102,894 ppm) and to validate the correlation between the electrochemical parameters (I_corr_, E_corr_ and E_pitt_) and ECM of Sn in the presence of Br^−^. Based on the in-situ video microscopy, ex-situ characterizations by scanning electron microscopy (SEM) coupled with energy dispersive X-ray spectroscopy (EDX) and X-ray photoelectron spectroscopy (XPS), the ECM mechanisms are proposed while the roles of precipitates and electrolyte flow during the ECM in the presence of sodium bromide (NaBr) are discussed. It is demonstrated that the ECM probability is linked to the synergistic effects of anodic dissolution rate, the presence of precipitates and the effects of convective liquid flow in the water droplet.

## Materials and methods

### Materials and setup for in-situ WDT

**Figure 1:**
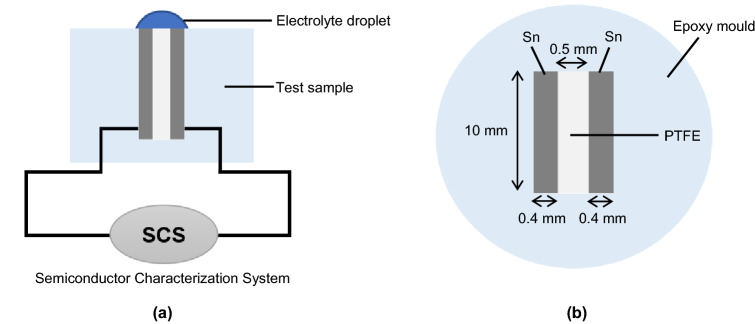
(**a**) Schematic of the WDT setup. (**b**) Top view of the test sample.

Two identical chemically pure (C.P.) Sn sheets with dimensions of 0.4 mm × 10 mm × 15 mm were aligned parallel and embedded in an epoxy resin mould. A 0.5 mm-thick polytetrafluoroethylene (PTFE) sheet was used as the spacer between two identical Sn foils as shown in Fig. 1. The total exposed area of the two pure Sn foils was 8 mm^2^. Copper wire was soldered at the back side of each Sn foil. The exposed surface of the Sn foils was polished using abrasive paper of 3,000 grit, then polished using the MD-Nap disk (Struers A/S) and lubricated by DiaPro Suspension Nap R1 (1 µm; Struers) to minimize the effects of surface roughness on the ECM process. Prior to the water drop tests (WDT), the Sn surfaces were rinsed in distilled water, degreased with isopropyl alcohol and dried in air.

The WDT was carried out using two-probe Semiconductor Characterization System (SCS) (Tektronix Keithley Model 4200-SCS) at ambient temperature (~ 25 °C). Sodium bromide (NaBr) solutions with concentrations of 1·10^−6^ M to 1.0 M were used as the electrolytes. During the WDT, 5 µL of NaBr electrolyte was drop-casted on the exposed surface, covering both Sn foils. A direct current (DC) with a bias voltage of 3 V was applied between the two Sn foils, as the anode and the cathode, respectively. The current flow through the electrodes and electrolytes was measured and recorded for 450 s. Simultaneously, a video of the whole WDT process was recorded in-situ under an optical microscope (MPI TS150). The TTF was determined based on the occurrence of the first current surge and the observation of dendritic bridges from the optical microscope.

### Ex-situ characterizations

The morphology of dendrites and precipitates formed during the tests were characterized using SEM coupled with EDX (Carl Zeiss, model no: Auriga) at an accelerating voltage of 15 kV. XPS was performed on the precipitates using ULVAC-PHI Quantera II system. The source of X-ray was Al Ka (1,486.6 eV) and the base pressure in the chamber was in the range of 10^−9^ mbar. The energy resolutions for the wide and narrow scans were 1 eV/step and 0.1 eV/step, respectively. The spectral deconvolution was performed by the MultiPak software. In order to eliminate the surface charging effects, calibration of the binding energy (BE) was done by referring to the carbon spectrum of C–C at 284.8 eV.

### Polarization test

The polarization tests were conducted using a potentiostat (Autolab, PGSTAT30, Netherlands). The counter electrode and reference electrode were a platinum (Pt) wire and a silver-silver chloride (Ag–AgCl), respectively. The working electrode was a pure Sn foil with an exposed area of 0.32 cm^2^. The polarization curves were measured in NaBr solutions with varying concentrations from 10^−6^ M to 1.0 M. The polarization test was performed using linear scan voltammetry from − 1.3 to + 1.3 V and at a scan rate of 1 mV/s. The results were analysed using general purpose electrochemical software (GPES).

### Statistical analyses

In order to obtain the statistical averages of the current flow and TTF, the WDT was repeated for ten times under each experimental condition. The means and standard errors of the current flow were calculated from ten replications. The ECM failure probability was calculated by dividing the total number of failures during the test by the total number of WDT (10) performed under the same conditions. The means and standard errors of TTF were then calculated based on the total number of samples failed during the tests.

## Results

### In-situ ECM investigations during WDT

**Figure 2:**
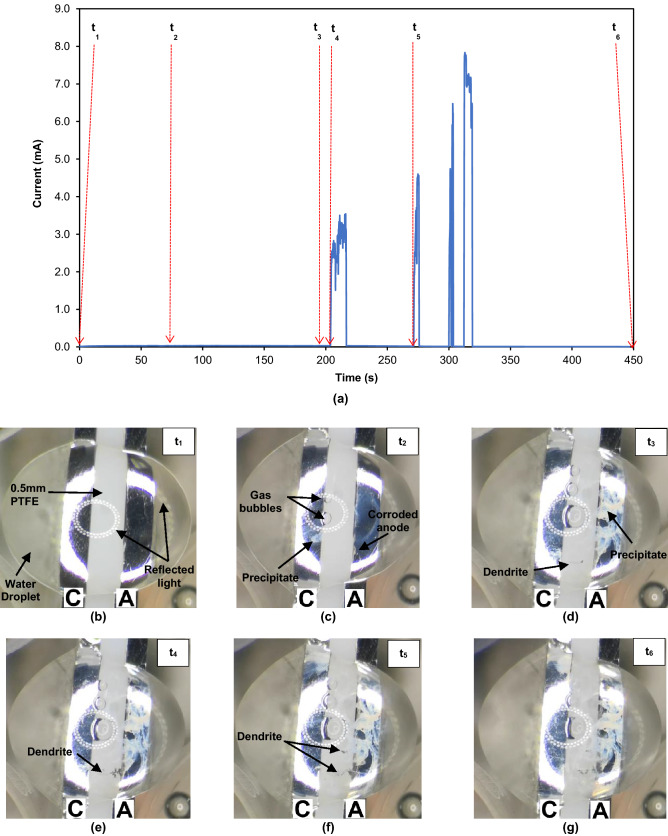
(**a**) Current vs. time curve obtained from the ECM test of Sn in 8·10^−4^ M NaBr and applied bias of 3 V and the corresponding in-situ microscopic images at: (**b**) 0 s; (**c**) 76 s; (**d**) 198 s; (**e**) 204 s; (**f**) 271 s; (**g**) 450 s (*C* cathode; *A* anode).

During the WDT, the ECM process of Sn in the presence of Br^−^ was observed in-situ under an optical microscope and recorded in a video (Supplementary Vid. S1). Figure 2 shows a typical current–time curve and the corresponding microscopic images recorded in-situ during the WDT, in 8·10^−4^ M NaBr solution, at an applied bias voltage of 3 V. Figure 2b–g presents the occurring events as a function of time during the WDT. The initial condition of the test sample surface upon the application of a 3 V bias potential (t_1_) is shown in Fig. 2b. A clean hemispherical water droplet is observed on the test sample that consists of two oppositely charged Sn electrodes. The presence of bright dotted rings on the surface of the water droplet is due to the reflected light from the microscope. At the early stages (t_1_ − t_2_) of ECM, white precipitates are formed in the NaBr electrolyte and gas bubbles are evolved at the cathode as shown in Fig. 2c. The amount of white precipitates gradually increases, while the bubbles grow continuously in size. The latter coalesces and eventually disintegrates with time. Simultaneously, Sn corrosion is observed from the change in the anode appearance from a shining metallic surface to a greyish surface. The dark dendrites in Fig. 2d nucleate on the cathode at 198 s (t_3_) and propagate for 6 s before bridging at 204 s (t_4_). When the dendritic bridging occurs between the oppositely charged electrodes at t_4_ (Fig. 2e), the current surges from 10^−3^ mA to ~ 3 mA (~ 1,000 times increment), confirming the short-circuiting between the two electrodes. The current drops to 10^−3^ mA after 12 s, indicating the collapse of the dendritic bridge between the anode and the cathode. During the WDT, the flow of liquid is observed in the static droplet. The liquid flow direction is illustrated in the Supplementary Fig. S1. The dendrites collapse due to the convective liquid flow of the electrolyte from the opposite direction or the burning off effects caused by the high current^1^. At 271 s (t_5_), the second peak was observed in the current–time curve (Fig. 2a) along with the second dendritic bridging (Fig. 2f).

The dendrites then collapse but are reconnected for a few times before the end of the WDT (t_6_). The time-to-failure (TTF) in this test is determined to be 204 s (t_4_) based on the occurrence of the first short circuit, which is confirmed from the observation of the dendritic bridging and the sudden current spike^33,34^. It is important to note that the gas bubbles and precipitate act as mechanical barriers to the growth of dendrites during the WDT.

**Figure 3:**
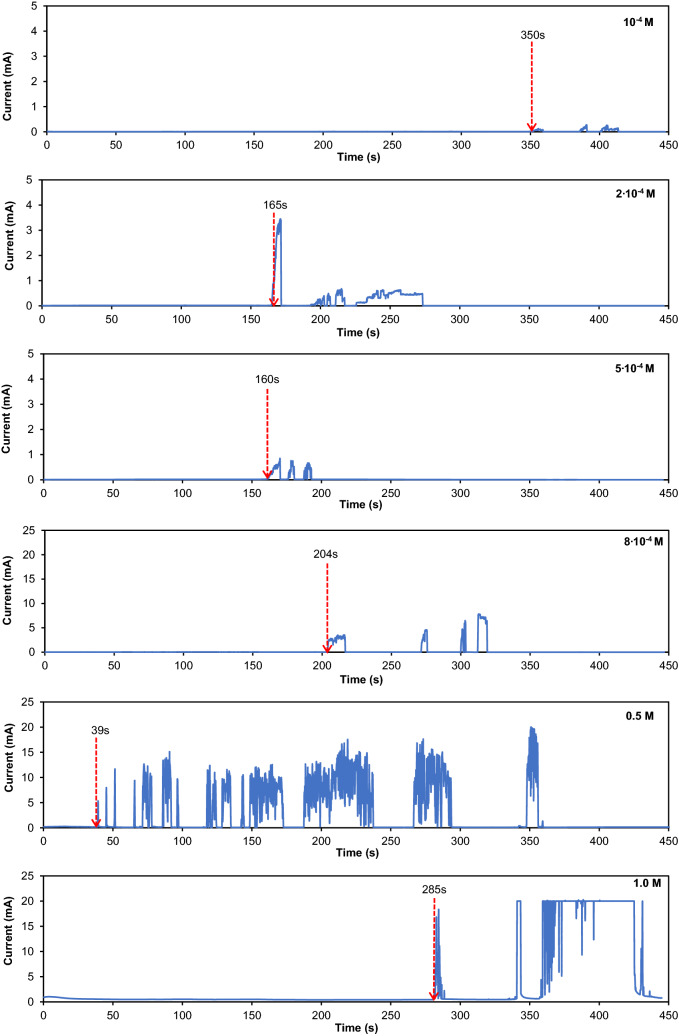
Current vs. time curves for the ECM of Sn at various Br^−^ concentrations and applied bias of 3 V. Red arrows pinpoint the time of the first current surge or the TTF.

Figure 3 shows a typical current–time response depicting the current surges in varying Br^−^ concentrations. In general, the time of the first current surge decreases with the Br^−^ concentrations. However, the time of the first current surge at the highest Br^−^ concentration (1.0 M) is longer than the time recorded in lower concentrations (2·10^−4^ M–0.5 M). This could be due to the strong effervescence and strong electrolyte convection which obstruct the dendritic propagation at the highest Br^−^ concentration. The current surges and drops for a few times in most cases, suggesting that the dendrites collapse and reconnect many times after the first bridging, indicating that more vigorous reactions occur at higher concentrations.

**Figure 4:**
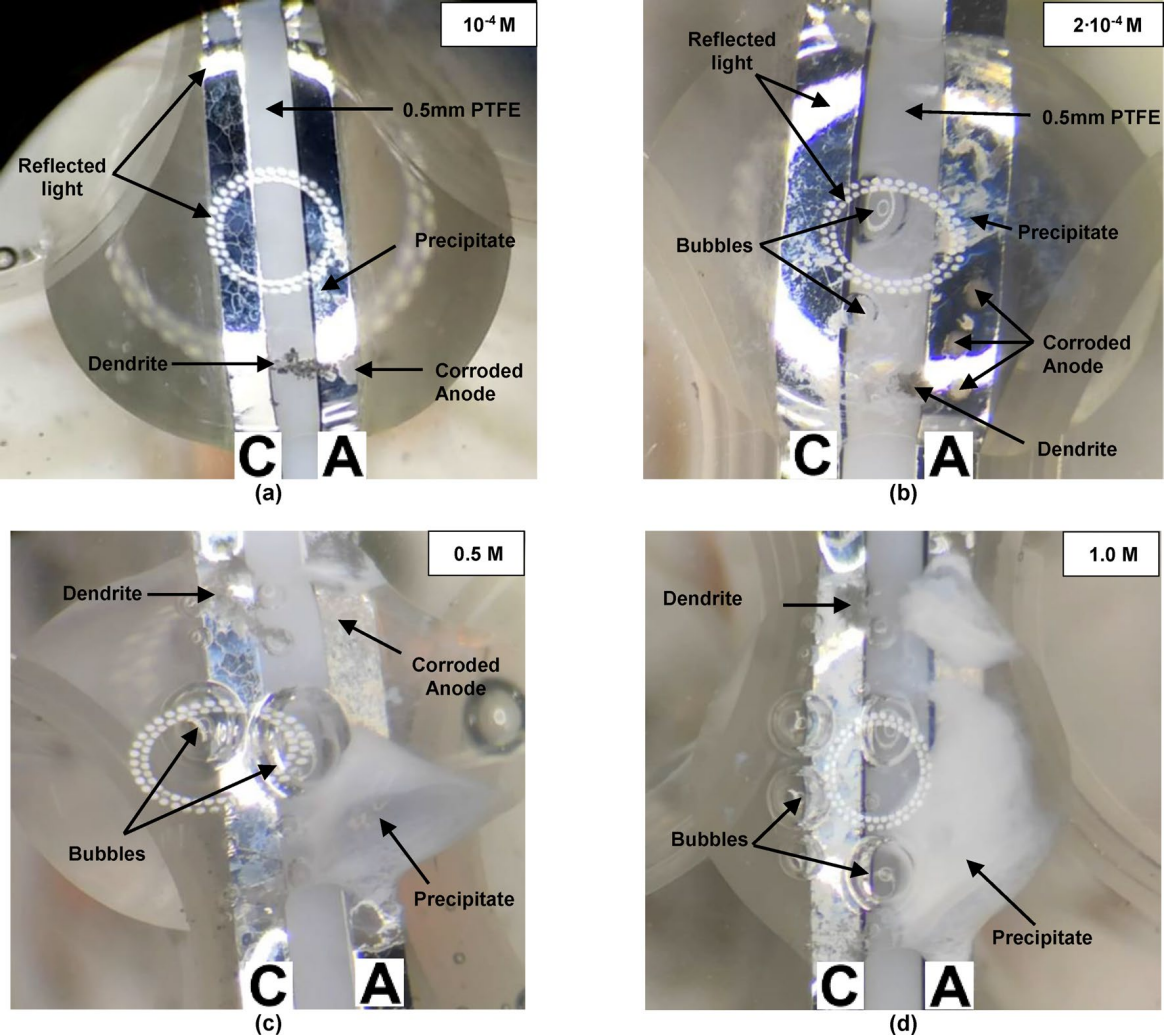
In-situ microscopic images of Sn electrodes at the end (450 s) of the ECM process at 3 V applied bias, in various Br^−^ concentrations: (**a**) 10^−4^ M; (**b**) 2·10^−4^ M; (**c**) 0.5 M and (**d**) 1.0 M (*C* cathode; *A* anode).

Figure 4a–d show the corresponding microscopic images acquired at the end of each WDT (450 s) at different Br^−^ concentrations. The amount of gas bubbles and precipitate formation increases with the Br^−^ concentration.

### Mean time-to-failure (MTTF) and probability of failure due to ECM

The in-situ study on ECM of Sn shows variations in the results, with some clear short-circuiting events whilst others do not, although the test parameters such as Br^−^ concentration and applied voltage are kept constant. The variations in the results reveal that the dendritic growth of Sn in the presence of Br^−^ is probabilistic in nature.

Table 1 summarizes the quantitative data obtained from ten repetitions of WDT in varying Br^−^ concentrations. In extremely low Br^−^ concentrations (10^−6^ M–10^−5^ M), the probability of failure is zero, as none of the 10 samples tested under the same condition showed any current surge or short-circuiting during the WDT. The test samples started to fail when the Br^−^ concentration is increased to 10^−4^ M, where 1 out of 10 samples suffers failure. The highest probability of failing occurs at intermediate Br^−^ concentration (2·10^−4^ M), where 6 out of 10 samples experience short-circuiting. A further increase in the Br^−^ concentration decreases the probability of the ECM failure. The samples did not short-circuit at concentration of 10^−3^ M–0.1 M. However, a still further increase in the concentration to 0.5 M and 1.0 M resulted in the ECM failure. Supplementary Figure S2 illustrates the trend of the ECM probability in a graphical form. In short, the probability of Sn failure due to the ECM initially increases but later decreases with the Br^−^ concentration, while a still further increase in the Br^−^ concentration enhances the risk of failure due to the ECM in the WDT.

**Table 1:** Probability of ECM failure and mean time-to-fail (MTTF) of Sn at various Br^−^ concentrations. Standard errors were calculated from the total number of failures during WDT.

Concentration of NaBr (M)	Probability of ECM failure	MTTF (s)
10^−6^	0/10	None
10^−5^	0/10	None
10^−4^	1/10	350.00
2 × 10^−4^	6/10	192.50 ± 49.08
5 × 10^−4^	3/10	137.29 ± 28.68
8 × 10^−4^	4/10	173.25 ± 21.61
10^−3^	0/10	None
10^−2^	0/10	None
0.1	0/10	None
0.5	1/10	39.00
1.0	5/10	287.04 ± 71.66

The average TTF or mean time-to-fail (MTTF) of the short-circuited samples are calculated and tabulated in Table 1. A higher MTTF implies a longer lifetime of the samples, while “none” indicates that short-circuiting does not occur at those concentrations throughout the WDT. The shortest and the longest MTTF recorded are 39 s (0.5 M) and 350 s (10^−4^ M), respectively.

### Ex-situ characterizations of dendrites and precipitate

**Figure 5:**
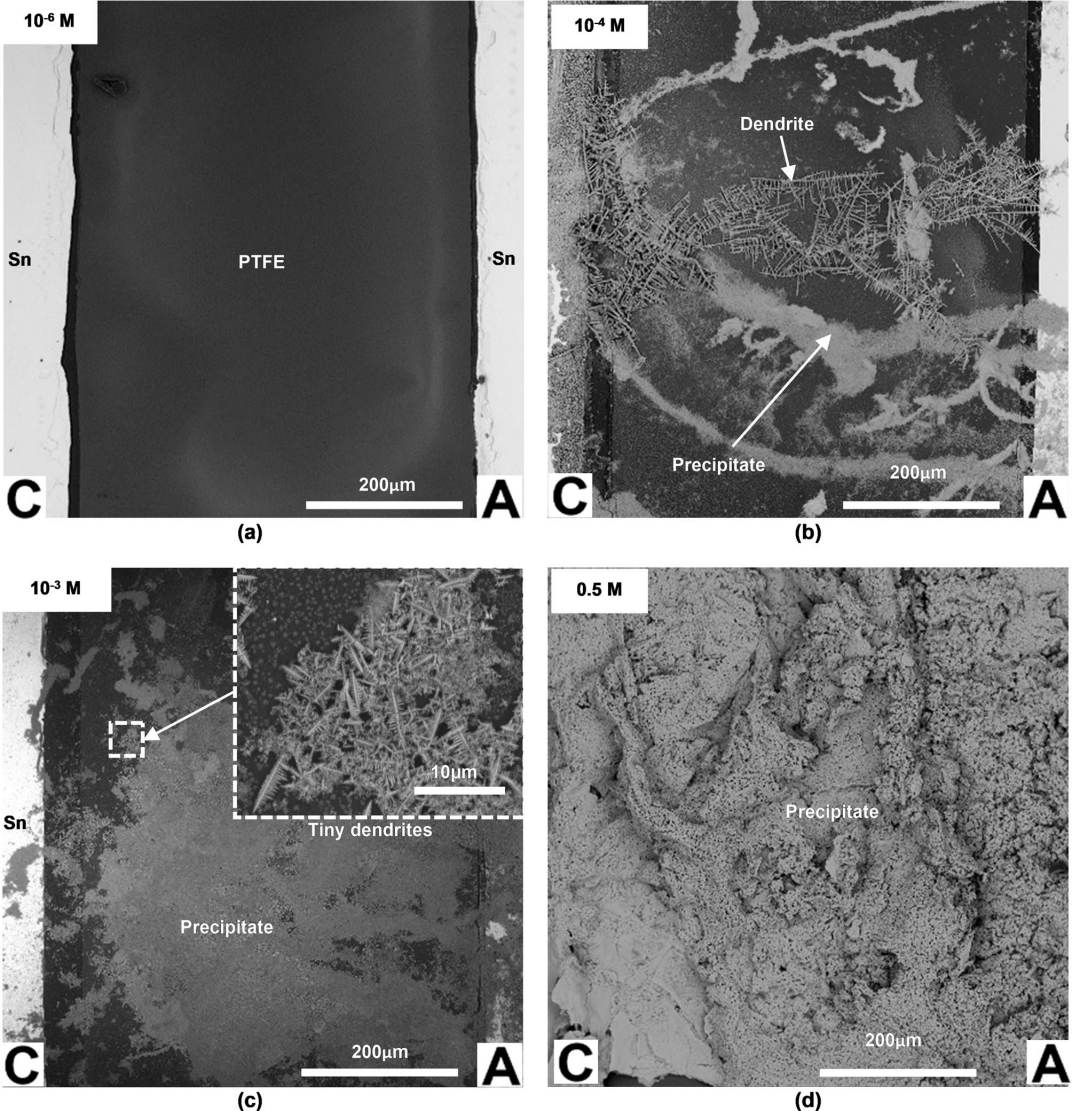
SEM images of the products formed at the area between two oppositely charged Sn electrodes after 450 s of WDT, at an applied bias voltage of 3 V, in electrolytes with various Br^−^ concentrations: (**a**) 10^−6^ M; (**b**) 10^−4^ M; (**c**) 10^−3^ M; (**d**) 0.5 M (*C* cathode; *A* anode).

Figure 5a–d presents the microstructures of the ECM products formed from the WDT in the presence of different Br^−^ concentrations. Figure 5a shows a clean sample surface at an extremely low Br^−^ concentration where no reaction products such as dendrites or precipitates are observed. When the concentration of Br^−^ is increased to 10^−4^ M, two types of reaction products are formed: long tree-like dendrites and precipitate (Fig. 5b). The dendrites are arranged in such a way that they connect the anode and the cathode. The dendrites with long branches are typically arranged at around right angle with respect to the main stem. The individual dendrite could grow around 175 μm in length while the precipitate is dense in appearance. A further increase in the Br^−^ concentration (10^−3^ M) results in the formation of massive precipitates (Fig. 5c). A higher magnification shows that the precipitate consists of many tiny dendrites (inset of Fig. 5c). These tiny dendrites found in the precipitate are around 7 μm in length. The amount of precipitate increases further at very high Br^−^ concentration (0.5 M).

The EDX analysis of the long dendrites formed in 2·10^−4^ M NaBr (see Supplementary Fig. S3) consists solely of Sn. The presence of fluorine (F) and carbon (C) peaks in the spectrum is attributed to the PTFE underneath the dendrite. It should be noted that the PTFE was used as the separator between the Sn electrodes. The presence of oxygen (O) peak could be due to the contamination caused by the precipitate. The EDX analysis of the precipitate formed in 0.5 M NaBr (see Supplementary Fig. S3) shows the presence of Sn (24.0 wt.%), O (18.1 wt.%), Na (9.8 wt.%) and Br (48.2 wt.%) elements.

**Figure 6:**
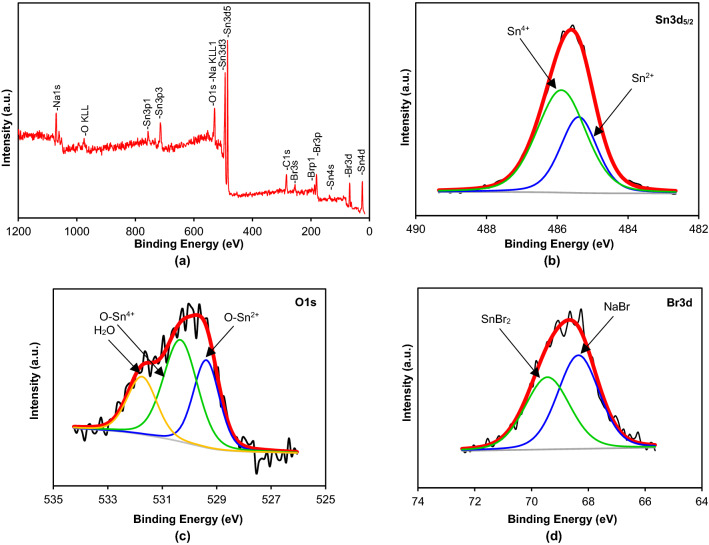
(**a**) XPS wide-scan spectrum; (**b**) XPS high resolution spectrum of Sn 3d_5/2_; (**c**) O 1 s and (**d**) Br 3d of precipitate formed during the ECM test of tin in the presence of 0.5 M NaBr at a bias voltage of 3 V.

XPS analysis was performed to investigate the chemical nature of the precipitate. Figure 6 shows the XPS spectra of the precipitate formed in 0.5 M NaBr. The XPS wide-scan spectrum (Fig. 6a) shows the presence of peaks belonging to C, O, Na, Br and Sn elements. The corresponding elemental composition obtained from the XPS results is shown in the Supplementary Table S1. The presence of C could be due to the contaminants from the experimental procedures. The presence of Sn, O, Na and Br suggests that the precipitate is a mixture of tin oxides and residue of NaBr salt.

The high-resolution spectra of Sn 3d_5/2_, O 1s and Br 3d are shown in Fig. 6b–d. The oxidation states of Sn in the precipitate are verified in Fig. 6b,c. The Sn 3d_5/2_ peak shown in Fig. 6b is composed of two Gaussian functions centred at about 485.35 eV and 486.05 eV, which corresponds to Sn2^+^ and Sn^4+^, respectively. The deconvolution of O 1s curve (Fig. 6c) shows three Gaussian functions centred at around 529.55 eV, 530.25 eV and 531.55 eV, which corresponds to the O-Sn^2+^, O-Sn^4+^ and water (H_2_O), respectively. These results confirm the presence of stannic compounds and stannous compounds in the precipitate. The amount of Sn^4+^ is slightly higher compared to Sn^2+^. The resolved components of Br 3d (Fig. 6d), centred around 69.55 eV and 68.25 eV, could be due to the SnBr_2_ and residual NaBr, respectively.

### Polarization curves

**Figure 7:**
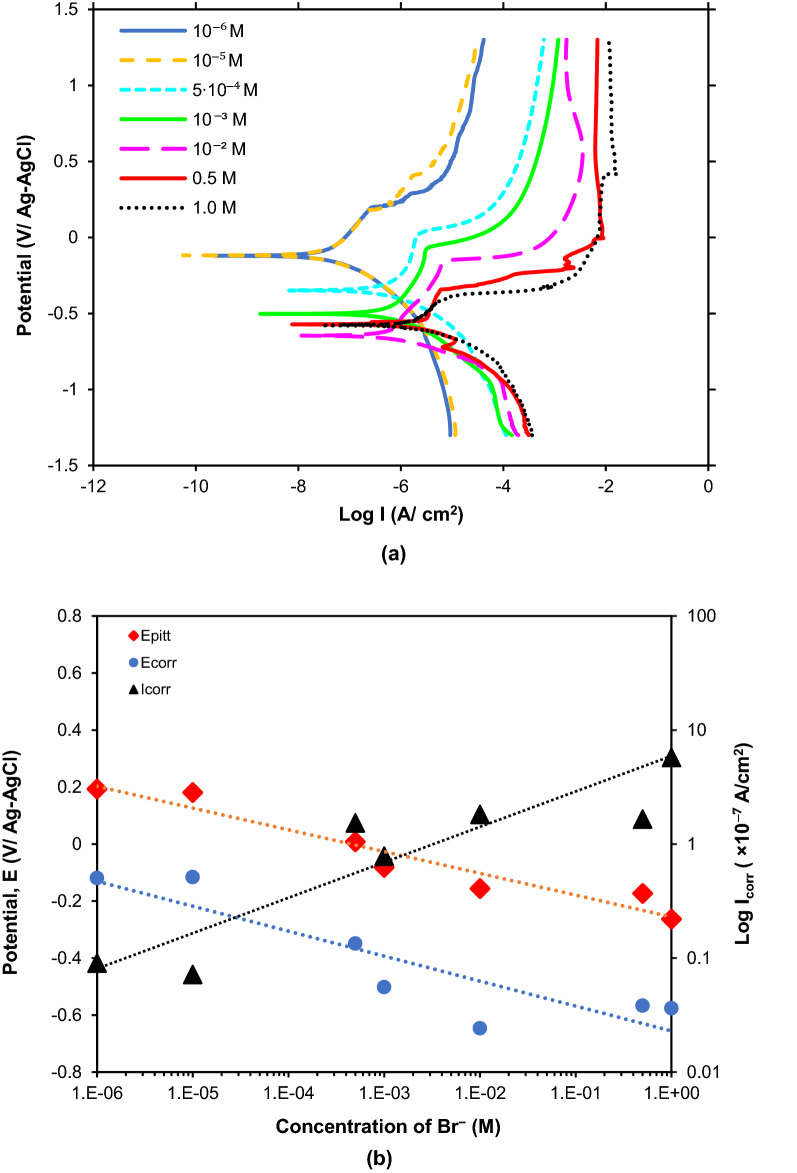
(**a**) Polarization curves of Sn in various Br^−^ concentrations. (**b**) Comparison of the electrochemical properties of pure tin in various Br^−^ concentrations.

Figure 7a shows the polarization curves of pure Sn in solutions containing various concentrations of Br^−^ ions (10^−6^ M–1.0 M) recorded at the ambient temperature. The values of E_corr_ and I_corr_ were calculated from the Tafel plots obtained from Fig. 7a. The E_pitt_ was also obtained from Fig. 7a from the onset of breakdown potential of the passivation. Figure 7b shows the relationship between the concentration of Br^−^ and the electrochemical parameters such as E_corr_, E_pitt_ and I_corr_. Generally, the E_corr_ and E_pitt_ decrease while the I_corr_ increases with the Br^−^ concentration.

## Discussion

It is found that pure Sn is susceptible to ECM in Br^−^ environment. Based on the in-situ investigation of WDT, the main reactions involved in the ECM of Sn include anodic dissolution of Sn, formation of white precipitates, evolution of gas bubbles at the cathode and growth of Sn dendrites at the cathode.

**Figure 8:**
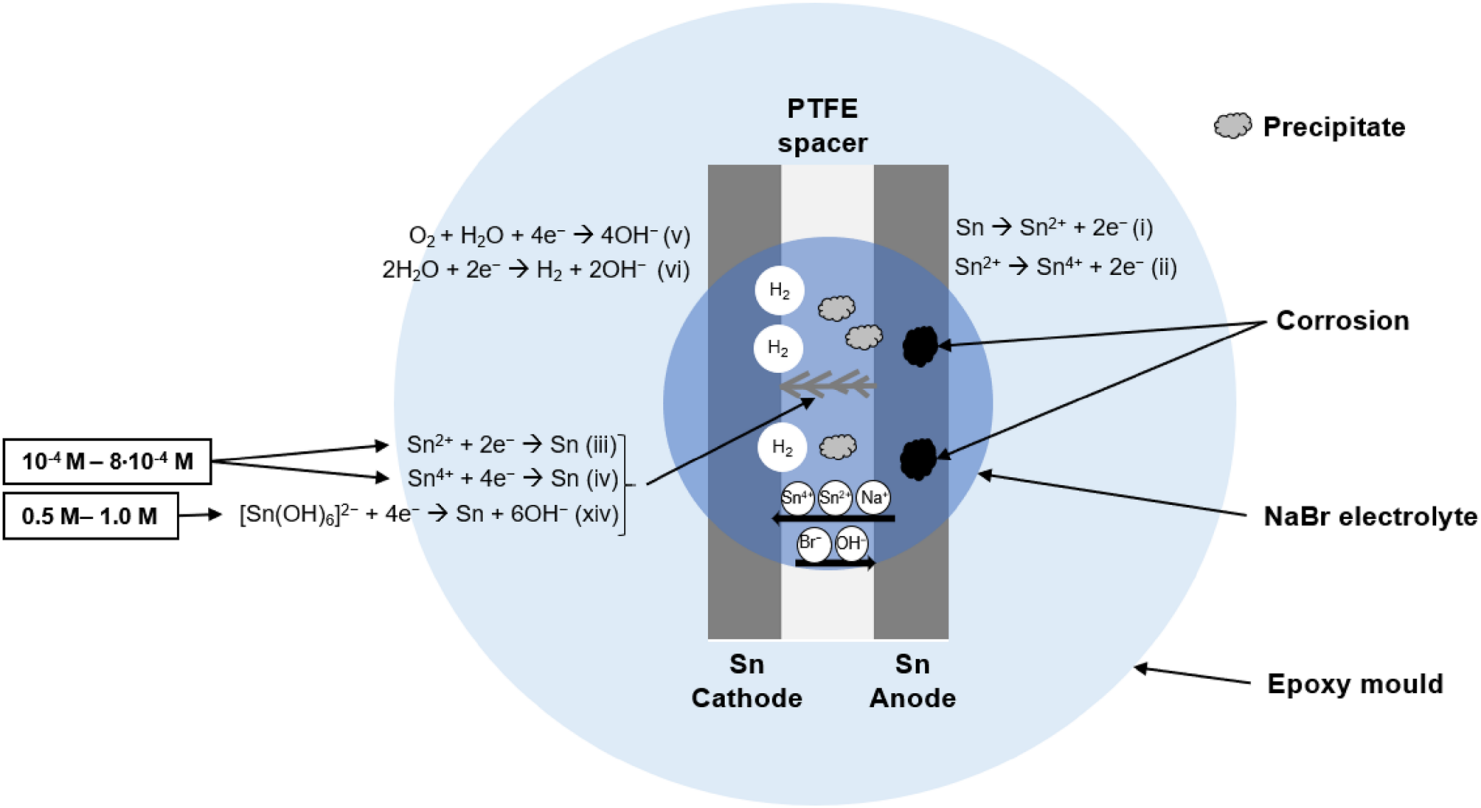
Schematic of plan view of electrochemical cell formed during WDT at applied bias of 3 V, in NaBr concentration of (not drawn to scale).

Based on the data obtained in this work and reaction mechanisms suggested in the literature^5,7,20,34,35^, the possible reaction steps that occur during the ECM of Sn are suggested below. Figure 8 illustrates the reactions involved during the ECM of Sn. In the presence of Br^−^, the ECM of Sn begins with the dissolution of the Sn anode to form Sn^2+^ ions as shown in (1), followed by the further oxidization of Sn^2+^ to form Sn^4+^ as shown in (2)^5,7,17,32,33^.

Reactions at the anode:

1 $$Sn\to {Sn}^{2+}+ {2e}^{-}$$

2 $${Sn}^{2+}\to {Sn}^{4+}+ {2e}^{-}$$

The anodic dissolution is followed by the transport of ions^5,7^. Positively charged Sn^2+^ and Sn^4+^ produced at the anode migrate towards the negatively charged Sn cathode due to ionic migration with the presence of the electrical field, the concentration gradient and electrolyte convection^7^. The Na^+^ released from NaBr is also attracted to the negative terminal. As Sn^2+^ or Sn^4+^ reaches the cathode, the discharge of Sn ions and deposition of metallic tin dendrites occur as described in (3) and (4)^5,7,17,20,35^.

Reactions at the cathode:

3 $${Sn}^{2+}+ {2e}^{-} \to Sn$$

4 $${Sn}^{4+}+ {4e}^{-} \to Sn$$

The reduction of dissolved O_2_ (5) in the electrolyte and the reduction of H_2_O (6) also occur at the cathode to release the hydroxide ions (OH^−^)^5,7,36,37^.

5 $${O}_{2}+ 2{H}_{2}O + {4e}^{-}\to {4OH}^{-}$$

6 $$2{H}_{2}O + {2e}^{-}\to {H}_{2}+ {2OH}^{-}$$

Reaction (6) is suggested as the main reaction compared to the oxygen reduction reaction as the concentration of dissolved oxygen (O_2_) is relatively low as compared to H_2_O in the electrolyte^7,20^. The evolution of gas bubbles at the cathode is due to the release of hydrogen gas (H_2_) as shown in (6).

Precipitation is observed during the in-situ investigation of WDT. The XPS results in Fig. 6 reveal the presence of stannous and stannic compounds. This is attributed to the formation of stannous hydroxide [Sn(OH)_2_] and stannic hydroxide [Sn(OH)_4_] from (7) and (8)^7,20^.

7 $${Sn}^{2+}+ {2OH}^{-} \to Sn{(OH)}_{2}$$

8 $${Sn}^{4+}+ {4OH}^{-} \to Sn{(OH)}_{4}$$

Based on the standard Pourbaix diagram of Sn, both Sn(OH)_2_ and Sn(OH)_4_ are stable compounds in neutral environment^37^. Owing to the low solubility product constant (K_sp_) of Sn(OH)_4_ (1.0·10^−5^^7^ at 25 °C)^7,38^ and Sn(OH)_2_ (5.45·10^−27^ at 25 °C)^38^, the white precipitates of Sn(OH)_4_ and Sn(OH)_2_ form rapidly upon the application of a bias voltage. Sn(OH)_4_ can also be formed from the direct oxidation of Sn anode or hydrolysis of Sn^4+^ as shown in reactions (9) and (10), respectively^5,7^.

9 $$Sn+4{H}_{2}O \to Sn{(OH)}_{4}+4{H}^{+}+ {4e}^{-}$$

10 $${Sn}^{4+}+4{H}_{2}O \to Sn{(OH)}_{4}+4{H}^{+}$$

In addition to the hydroxides, XPS data also reveal the presence of NaBr and SnBr_2_ (Fig. 6d) in the precipitate. The presence of NaBr is attributed to salt crystallization from the evaporation of water, as the samples were left to dry in the air before the ex-situ characterizations. Based on the thermodynamics data, SnBr_2_ (∆_f_G^0^_m_ =  − 237.9 kJ·mol^−1^ at 298.15 K)^39^ and SnBr_4_ (∆_f_G^0^_m_ =  − 359.5 kJ·mol^−1^ at 298.15 K)^39^ can form from (xi) and (xii), as the Sn^2+^, Sn^4+^ and Br^−^ migrate and encounter each other under the influence of an electric field.

The formation of SnBr_2_ and SnBr_4_ are as follows:

11 $${Sn}^{2+}+ 2{Br}^{-} \to Sn{Br}_{2}$$

12 $${Sn}^{4+}+ 4{Br}^{-} \to Sn{Br}_{4}$$

SnBr_4_, however, was not detected in the XPS spectrum (Fig. 6d) due to the insufficient database.

It should be noted that tiny dendrites (~ 7 µm in length) are observed in the precipitate (Fig. 5c). These are in addition to the longer Sn dendrites (~ 175 µm) which are electrically conductive and are responsible for the short-circuiting failure. The presence of tiny dendrites was also reported in the ECM of Sn-2Pb in the presence of 10 ppm NaCl^33^. By using transmission electron microscopy (TEM) and the electron diffraction patterns, it was confirmed that these tiny dendrites are tin hydroxides. Thus, it is suggested that the small dendrites observed in this work are also tin hydroxides.

It is suggested that the mechanism of Sn dendritic growth changes at a very high Br^−^ concentration (0.5 M–1.0 M). It was reported that in highly alkaline conditions, further reaction of Sn(OH)_4_ with OH^−^ forms the complex ion [Sn(OH)_6_]^2−^ as shown in (13)^20,40^.

13 $$Sn{(OH)}_{4}+2O{H}^{-}\to {[Sn{\left(OH\right)}_{6}]}^{2-}+ 4{H}_{2}O$$

At high Br^−^ concentrations (≥ 0.5 M NaBr), excessive OH^−^ is produced at the cathode and thus creates a highly alkaline environment in the vicinity of the cathode (14). The migration of the OH^−^ to the anode causes pitting corrosion and strips the hydroxide layer from the anode (13) which releases the [Sn(OH)_6_]^2−^ complex ion into the solution. The [Sn(OH)_6_]^2−^ complex ion is then reduced to form the Sn dendrites as shown in (14)^5,7,20,40^.

14 $${[Sn{\left(OH\right)}_{6}]}^{2-}+ 4{e}^{-}\to Sn+6O{H}^{-}$$

This reaction is a widely accepted mechanism for the alkaline plating process used in industry^40^.

The effect of Br^−^ concentration in the ECM of Sn was investigated in a wide range of concentrations (10^−6^ M–1.0 M). The results show that the rate of reactions, corrosion properties and probability to ECM failure of Sn are influenced by the Br^−^ concentration.

**Figure 9:**
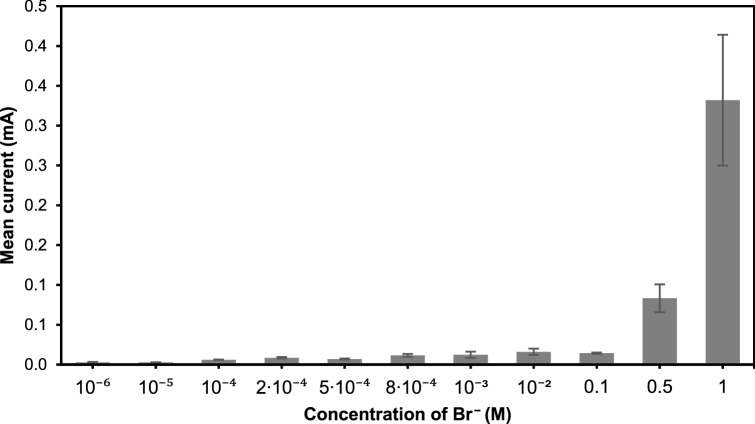
Mean current measured during the first 10 s of WDT. Error bars indicate the standard errors of the means calculated from 10 replications.

The effects of different NaBr concentration were investigated using the in-situ observation of WDT and polarization tests. It is obvious that the ECM reactions such as the corrosion of anode, formation of precipitates and evolution of gas bubbles at the cathode, are more vigorous with increasing Br^−^ concentration. The average current in the first 10 s during the WDT increases significantly with the Br^−^ concentration (Fig. 9). This is related to the increased amount of electrically charged species^41^. Since NaBr solution is a strong electrolyte that dissociates completely in water to form Na^+^ and Br^−^, the conductivity of NaBr electrolyte increases with the number of ions dissolved in the solution with the NaBr concentration^15,42^. Therefore, the reaction rate increases with the solution concentration, due to the increase in the conductivity of the electrolyte.

The increase in the rate of reaction with Br^−^ concentration is also revealed in the polarization curves. Figure 7b shows that the increase in Br^−^ concentration obviously increases the I_corr_ and therefore accelerates the anodic dissolution. Concurrently, E_corr_ and E_pitt_ show an opposite trend where E_pitt_ represents the breakdown of the passivation film on the Sn metal^43,44^. The increase in Br^−^ concentration leads to the decrease in the E_pitt_, therefore facilitates the dissolution of the passivation film on the Sn surface. This indicates that pure Sn is more prone to corrosion in higher Br^−^ concentration.

Despite the accelerated reactions at higher Br^−^ concentrations, the probability of short-circuiting due to the ECM dendritic growth does not increase monotonously with the Br^−^ concentration. The dendritic growth and consequently the short-circuiting events do not depend only on the corrosion properties such as E_corr_, E_pitt_ and I_corr_, but rather on the synergistic interplay between different factors involved. Figure S2 illustrates the experimentally determined probability as a function of Br^−^ concentration. Overall, the probability of short-circuiting initially increases (10^−6^ M–2·10^−4^ M) but later decreases (2·10^−4^–0.1 M) with the Br^−^ concentration. The probability of short-circuiting increases again upon further increase in the Br^−^ concentration (≥ 0.5 M).

When the Br^−^ concentration is extremely low (≤ 1·10^−5^ M), the Sn dendritic growth is not initiated as the rate of reaction is extremely slow due to the very low solution conductivity. The increase in the Br^−^ concentration results in an accelerated dissolution of the Sn anode, which in turn promotes the feasibility of ECM. The probability is thus increased at intermediate Br^−^ concentrations (2·10^−4^ M–8·10^−4^ M). The highest probability was found at 2·10^−4^ M. The precipitate formation is likewise accelerated with more metal ions and hydroxyl ions being generated at increased Br^−^ concentration. The massive precipitate formed at higher concentrations (10^−3^ M–0.1 M) between the electrodes acts as barrier to the migration of charged ions^20^. Therefore, only accelerated corrosion without the formation of Sn dendrites is observed at higher concentrations (10^−3^ M–0.1 M) under the optical microscope. A further increase in the Br^−^ concentrations to 0.5 M leads to the formation of a highly alkaline environment. This results in the re-dissolution of the stannic hydroxide precipitates to form the [Sn(OH)_6_]^2−^ complex ion as shown in (xiii). The complex ions are then further reduced to form the Sn dendrites at the cathode.

Minzari et al.^5^ reported the probability of ECM of Sn in the presence of 10–250 ppm (8.4·10^−5^ M–2·10^−4^ M) Br^−^. The probability of Sn dendritic growth was found distributed normally around a bell curve as a function of the anodic dissolution rate. It was suggested that the highest probability of dendritic formation occurs at an optimum anodic dissolution rate. The result obtained in this work at Br^−^ concentrations lower than 0.5 M agrees with the trend observed in the previous work^5^. However, at 0.5 M, it is observed that the ECM failure probability increases again from zero to 0.1. This could be attributed to the re-dissolution of precipitates which ceases to act as barrier against Sn dendritic growth. Zhong et al.^17^ investigated the effects of varying Cl^−^ concentrations (10^−4^ M–0.5 M) on the ECM of Sn. They found that the Sn dendrites propagated in low and high concentrations (≤ 0.005 M and ≥ 0.07 M), while the dendritic growth was hindered at intermediate Cl^−^ concentrations (0.01 M–0.03 M) because the precipitate acts as a spatial barrier that hinders the migration of ions. The mechanisms of the dendritic growth reported are related to the direct reduction of Sn^2+^ and Sn^4+^ ions at less than 0.005 M NaCl and the reduction of [Sn(OH)]^2−^ at concentrations higher than 0.07 M Cl^−^. The similarities between the findings by Zhong et al.^17^ involving Cl^−^and Br^−^ in this work show that the ECM of Sn in the presence of halide contaminants in general shares common mechanisms.

Further investigations were carried out in order to compare the ECM susceptibility of Sn in the presence of Br^−^ to that of Cl^−^. The WDTs were conducted using the same test configurations, except that the electrolytes were changed to 10^−4^ M–10^−2^ M NaCl. The results are recorded in Table 2. It is observed that the probability to ECM failure of Sn in the presence of Cl^−^ is much higher compared to that in Br^−^. Almost all test samples experienced dendritic growth and short-circuiting in the presence of Cl^−^. While 10^−2^ M and 10^−3^ M Br^−^ resulted in zero ECM failure, the presence of 10^−2^ M and 10^−3^ M Cl^−^ resulted in 100% failure. The MTTF in 10^−4^ M Cl^−^ (~ 215 s) is lower than that in the presence of 10^−4^ Br^−^ (350 s). The results of ECM probability and MTTF indicate that Cl^−^ has a higher tendency to cause ECM in Sn compared to Br^−^.

**Table 2:** Probability of ECM failure and mean time-to-fail (MTTF) of Sn at various Cl^−^ concentrations. Standard errors were calculated from the total number of failures during WDT.

Concentration of NaCl (M)	Probability of ECM failure	MTTF (s)
10^−4^	4/5	215.28 ± 86.26
10^−3^	5/5	45.77 ± 19.76
10^−2^	5/5	145.49 ± 24.22

Although the ECM mechanisms of Sn in the presence of Br^−^ and Cl^−^ are the same, their aggressiveness towards the corrosion of Sn are different. In general, Cl^−^ is more aggressive than Br^−^ in the corrosion of a metal as Br^−^ is a weaker base compared to Cl^−^^45–47^. This is attributed to the smaller halide ion radius and the smaller atomic mass of Cl^−^, which lead to the increased aggressiveness^45^.

The results in the literature^21–25^ suggest that the probability of the Sn dendritic growth is related to the E_pitt_ and I_corr_ which determines the corrosion of Sn. However, no such relationship is observed in this work, the reason of which is explained above.

The convection inside the water droplet affects the ECM of Sn significantly in the condensed ECM. During the WDT, the liquid flow is from the cathode to the anode at the centre of droplet but bounces back from the anode to the cathode at the droplet boundary (Supplementary Vid. S1, 00:35 to 00:47). The liquid flow is attributed to the natural convection and forced convection^48^. Natural convection arises from the concentration gradients of the ionic species^48,49^, while forced convection is commonly related to the evolution of hydrogen gas bubbles and its consequent collapse after coalescence^7,48^. The convection could be also due to the evaporation of the electrolyte^50^. The loss of water was observed from the reduction of droplet thickness after performing the WDT for 450 s (see Supplementary Fig. S5).

The fluid motion inside a water droplet is one of the mechanisms that promotes the ion transportation from the anode to the cathode and vice versa, apart from the electrical field and concentration gradient^7,51^. The convection process accelerates or decelerates the dendritic growth during the ECM, depending on the direction of the liquid flow. Since the growth location of dendrites on the cathode occurs randomly, there is a large deviation in the MTTF (Table 1). The time of the dendritic propagation is shortened if the dendrites grow at the location where the liquid flow is from the cathode to the anode but is lengthen when the dendritic growth is against the direction of the liquid flow. The turbulent flow of electrolytes could dislodge the bridged dendrites (Supplementary Vid. S1, 01:24 to 01:37), causing the current to drop dramatically in the current versus time graphs obtained during the WDT. The convection inside the electrolyte droplet plays an important role in the condensed ECM, and therefore should not be neglected.

## Conclusion

The effects of Br^−^ concentration (1·10^−6^ M to 1.0 M) on the ECM behaviour of Sn were investigated. Overall, the reactions during ECM such as the corrosion of anode, formation of precipitates and evolution of gas bubbles are more vigorous at higher Br^−^ concentrations. The polarization curves showed that I_corr_ of Sn increases, while the E_corr_ and E_pitt_ of Sn decrease with Br^−^ concentration from 10^−6^ M to 1.0 M. However, the probability to ECM failure is not related to the electrochemical properties. The probability of ECM failure initially follows a bell-curve trend, where short circuit occurred at intermediate Br^−^ concentration range (10^−4^ M–8·10^−4^ M). Lower Br^−^ concentrations (≤ 10^−5^ M) did not show dendritic nucleation, while higher Br^−^ concentrations (10^−3^ M–0.1 M) lead to the formation of excessive precipitates which hinder dendritic growth. The further increase in Br^−^ concentration (≥ 0.5 M) leads to the re-dissolution of precipitate to form the [Sn(OH)_6_]^2−^ complex ions, followed by the reduction of the complex ions to form the dendrites. The presence of physical factors such as excessive precipitates, gas bubbles and fluid motion in the electrolyte outweigh the influence of the electrochemical properties in the ECM of Sn in the presence of Br^−^ contaminant.

## Supplementary Information

Supplementary Information 1.

Supplementary Video 1.
