# Supplementary material for: In-situ study of electrochemical migration of tin in the presence of bromide ion
Source: Sci Rep. 2021 Aug 3;11:15768. doi: 10.1038/s41598-021-95276-0 (PMC8333333; doi:10.1038/s41598-021-95276-0)
Supplement: Supplementary file 1 — Supplementary Information 1. [file 41598_2021_95276_MOESM1_ESM.pdf]

Supplementary Information

In-situ study of electrochemical migration of tin in the presence of bromide ion

Ee Lynn Lee[1,2*], A. S. M. A. Haseeb[1,2*], Wan Jeffrey Basirun[3,4], Yew Hoong Wong[1,2], Mohd Faizul Mohd Sabri[1,5] & Boon Yew Low[6]

[1]Department of Mechanical Engineering, Faculty of Engineering, Universiti Malaya, 50603 Kuala Lumpur, Malaysia.

[2]Centre of Advanced Materials, Faculty of Engineering, Universiti Malaya, 50603 Kuala Lumpur, Malaysia.

[3]Department of Chemistry, Faculty of Science, Universiti Malaya, 50603 Kuala Lumpur, Malaysia.

[4]Nanotechnology & Catalysis Research Centre (NANOCAT), Institute for Advanced Studies, Universiti Malaya, 50603 Kuala Lumpur, Malaysia.

[5]Centre for Energy Sciences, Faculty of Engineering, Universiti Malaya, 50603 Kuala Lumpur, Malaysia.

[6]Process Innovation, NXP Malaysia Sdn. Bhd., 47300 Petaling Jaya, Selangor, Malaysia.

*email: eelynn@um.edu.my; haseeb@um.edu.my

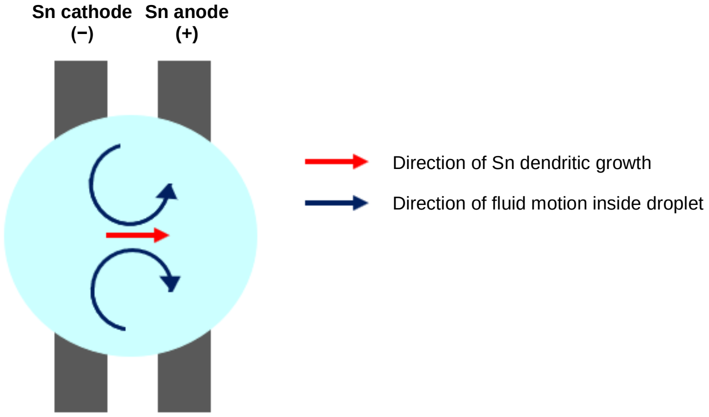

Supplementary Figure S1. Schematic of electrolyte flow (top view) when 3 V bias voltage is applied.

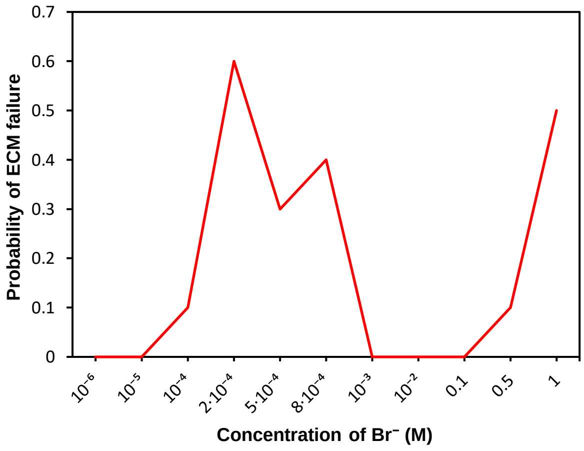

Supplementary Figure S2. Probability of short-circuiting as a function of Br^{-} concentration.

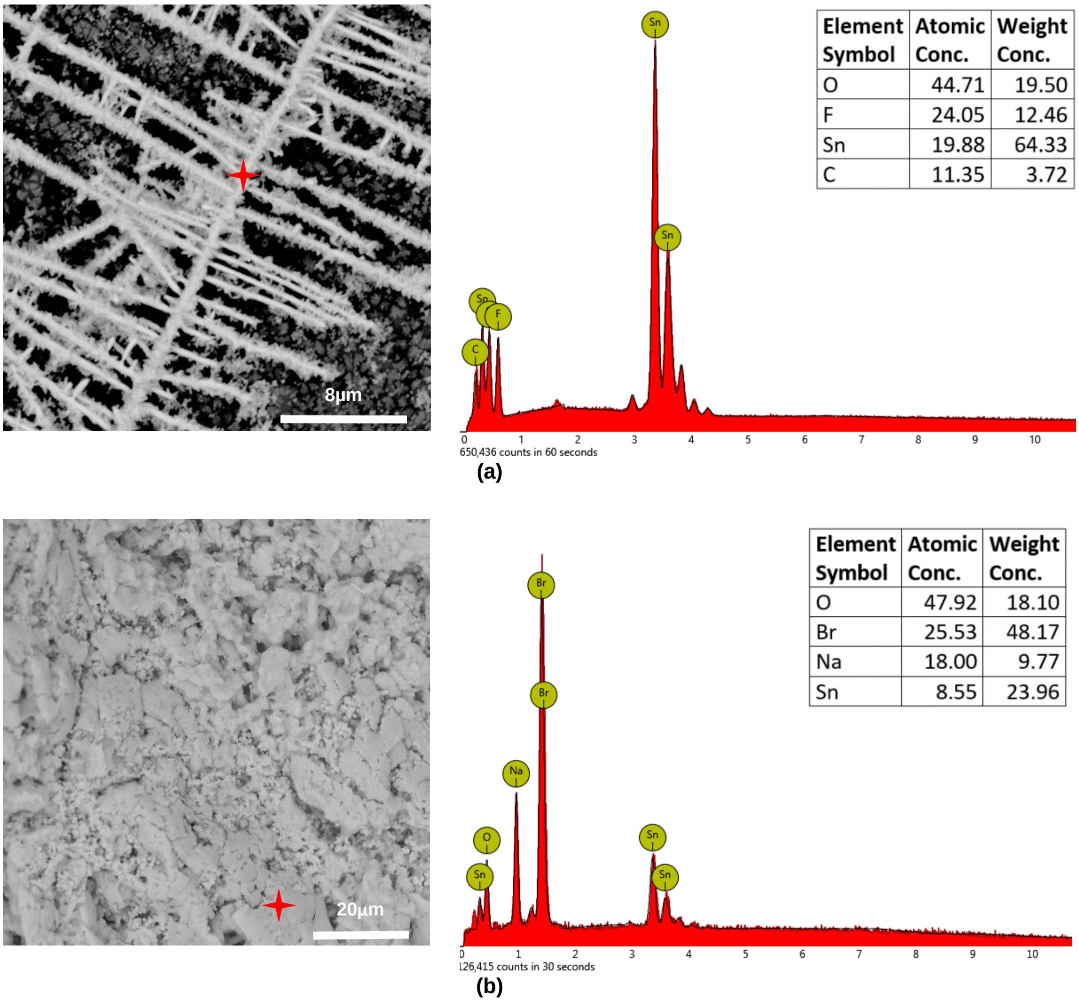

Element Symbol	Atomic Conc.	Weight Conc.
O	44.71	19.50
F	24.05	12.46
Sn	19.88	64.33
C	11.35	3.72

Element Symbol	Atomic Conc.	Weight Conc.
O	47.92	18.10
Br	25.53	48.17
Na	18.00	9.77
Sn	8.55	23.96

Supplementary Figure S3. SEM image and its respective EDX spectrum of (a) Sn dendrite (in the presence of $2 \cdot 10^{-4}$ M NaBr, 3 V). (b) Precipitate (in the presence of 0.5 M NaBr, 3 V).

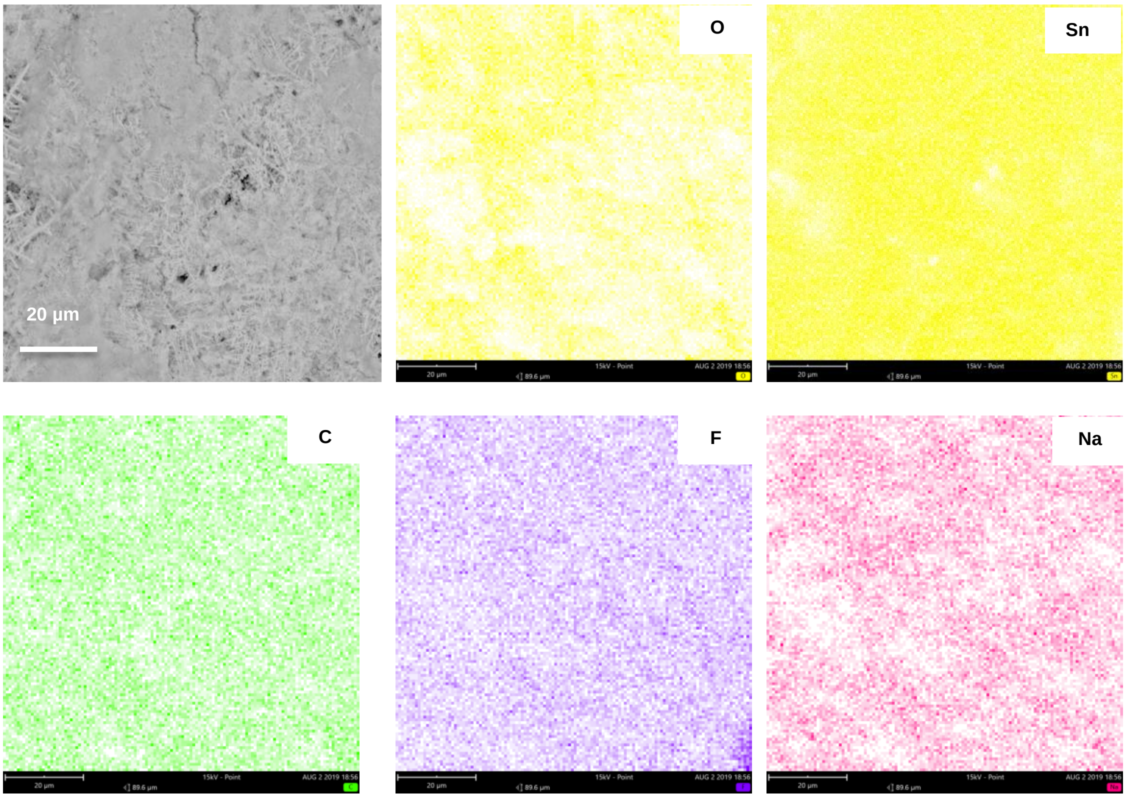

Supplementary Figure S4. EDX mapping showing the composition of the products formed at the area between two oppositely charged Sn electrodes after 450 s of WDT, at an applied bias voltage of 3 V, in the presence of $2{\cdot}10^{-4}$ M Br^{-}.

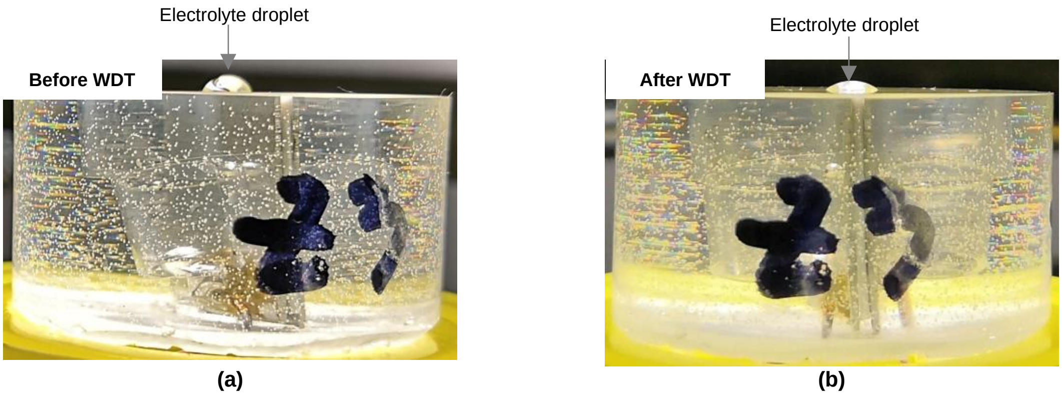

Supplementary Figure S5. Front view of a typical test sample showing the thickness of NaBr electrolyte droplet (a) before the WDT at 0 s and (b) after the WDT at 450 s.

Element	C 1s	O 1s	Na 1s	Br 3d	Sn 3d
At%	39.01	25.77	15.48	8.56	11.19

Supplementary Table S1. Elemental composition obtained from the XPS results for the precipitates formed in the presence of 0.5 M NaBr and in an applied bias voltage of 3 V.
